# Overexpression of microRNA-722 fine-tunes neutrophilic inflammation by inhibiting *Rac2* in zebrafish

**DOI:** 10.1242/dmm.030791

**Published:** 2017-11-01

**Authors:** Alan Y. Hsu, Decheng Wang, Theodore Gurol, Wenqing Zhou, Xiaoguang Zhu, Hsiu-Yi Lu, Qing Deng

**Affiliations:** 1 Department of Biological Sciences, Purdue University, West Lafayette, IN 47907, USA; 2 Purdue Institute for Inflammation, Immunology and Infectious Disease, Purdue University, West Lafayette, IN 47907, USA; 3 Purdue University Center for Cancer Research, Purdue University, West Lafayette, IN 47907, USA

**Keywords:** Zebrafish, MicroRNA, miRNA, Systemic inflammation, Cell motility

## Abstract

Neutrophilic inflammation is essential for defending against invading pathogens, but can also be detrimental in many clinical settings. The hematopoietic-specific small Rho-GTPase Rac2 regulates multiple pathways that are essential for neutrophil activation, including adhesion, migration, degranulation and production of reactive oxygen species. This study tested the hypothesis that partially suppressing *rac2* in zebrafish neutrophils by using a microRNA (miRNA) would inhibit neutrophil migration and activation, which would reduce the immunological damage caused by systemic inflammation. We have generated a transgenic zebrafish line that overexpresses microRNA-722 (miR-722) in neutrophils. Neutrophil motility and chemotaxis to tissue injury or infection are significantly reduced in this line. miR-722 downregulates the transcript level of *rac2* through binding to seed-matching sequence in the *rac2* 3′UTR. Furthermore, miR-722-overexpressing larvae display improved outcomes in both sterile and bacterial systemic models, which correlates with a robust upregulation of the anti-inflammatory cytokines in the whole larvae and isolated neutrophils. Finally, an miR-722 mimic protects zebrafish from lethal lipopolysaccharide challenge. Together, these results provide evidence for and the mechanism of an anti-inflammatory miRNA that restrains detrimental systemic inflammation.

## Abstract (precis)

**Summary:** Identification of a microRNA that suppresses Rac2 expression and regulates neutrophil migration and systemic inflammation. This article has an associated First Person interview with the first author of the paper as part of the supplementary information.

## INTRODUCTION

How to dampen immune activation is a major challenge in modern medicine. Neutrophils are the most abundant white blood cells in the circulation and the first line of defense against infections. Although essential for battling against pathogens, acute or chronic neutrophil activation drives immunopathology in numerous human diseases, including those directly involving an immune component (such as organ transplantation, sepsis and rheumatoid arthritis) and those that are not obviously linked (such as diabetes, neurodegenerative disease and cancer) (Borregaard, 2010; Nathan, 2006). Neutrophils release toxic granular contents, including proteases and extracellular traps, and produce a large amount of reactive oxygen species, which help with eliminating the threat of pathogens but can cause detrimental effects on the host. Recent evidence suggests that neutrophils, in addition to mediating acute inflammation, are a critical regulator of the inflammatory landscape. They live longer than previously recognized (Pillay et al., 2010). In addition, they initiate (Sreeramkumar et al., 2014), disseminate (Woodfin et al., 2011) and critically regulate the magnitude of inflammation (Warnatsch et al., 2015) while bridging innate and adaptive immunity (Abi Abdallah et al., 2011; Lim et al., 2015) in both sterile inflammation and infection. Thus, a successful strategy to prevent the infiltration of neutrophils is expected to significantly improve inflammatory conditions and reduce the risk of many modern diseases. As such, the microtubule-destabilizing agent colchicine, a potent inhibitor of neutrophil motility and activation, is approved for treating acute inflammation in familial Mediterranean fever and gout patients (Cocco et al., 2010). However, colchicine and the broad-spectrum anti-inflammatory agent corticosteroids lack neutrophil specificity and are inevitably accompanied by adverse side effects (Cocco et al., 2010). There is an urgent need to develop anti-neutrophil therapies that would benefit a diverse population suffering from inflammatory ailments.

MicroRNAs (miRNAs) are evolutionarily conserved, small non-coding RNAs that post-transcriptionally regulate protein synthesis (Fabian and Sonenberg, 2012). miRNA expression is controlled by specific promoters and regulated at the transcriptional and post-transcriptional levels. The long primary transcripts are processed in the nucleus into short ∼70 nt precursor miRNA hairpins, and further exported and processed in the cytoplasm into mature ∼22 bp miRNA duplexes, containing both 5p and 3p strands. The duplex is then loaded into Argonaut (AGO) proteins, where the passenger strand is degraded, thus allowing the guide strand to direct the miRNA-induced silencing complexes to partial-complementary target sites (Gurol et al., 2016). Either or both of the 5p and 3p strands can act as the predominant functional strand, depending on the unstable terminus at the 5′ end or other unknown mechanisms. The majority of the miRNAs bind to their target transcripts through complementarity in the 3′UTR to suppress gene expression, although, in some cases, enhanced target gene expression was observed (Ma et al., 2010). The seed sequence (positions 2-8 of the mature miRNA) is the major determinant of target recognition, although the contribution of other nucleotides cannot be excluded (Helwak et al., 2013). As of October 2017, 2588 human mature miRNAs have been identified and indexed in miRbase (www.mirbase.org), which are implicated in a wide variety of cellular processes and human diseases. miRNAs and anti-miRNAs are recent additions to the clinician's arsenal as next-generation therapeutics to treat human diseases (Broderick and Zamore, 2011; Hayes et al., 2014). Currently, miR-122 antagonists are in clinical trials for chronic hepatitis C infection (RG-101 by Regulus). Extensive effort has been made characterizing inflammation-related miRNAs. The miRNA expression profiles in various inflammatory conditions, including sepsis, have been documented (Gurol et al., 2016). However, the use of this information is currently limited to establishing miRNAs as biomarkers and diagnostic tools. The biological functions of these miRNAs and their therapeutic potential are merely starting to emerge. The majority of miRNA inflammation research is restricted to macrophages, with their roles in neutrophils being poorly characterized. Human peripheral blood neutrophils (Gantier, 2013; Landgraf et al., 2007; Ward et al., 2011) and activated tissue-infiltrating neutrophils (Larsen et al., 2013) each express a different profile of miRNAs. It is reasonable to speculate that miRNAs are potent regulators of neutrophil function and inflammation.

In the present study, we aim to identify a miRNA that would restrain hyperactive neutrophilic inflammation and to test its impact in acute systemic inflammation settings. Rac2, a member of the Rho small-GTPase family, is restricted to the hematopoietic lineage, which plays a principal role in regulating the actin cytoskeleton and neutrophil biology. By using neutrophils isolated from *Rac2*-knockout mice (Roberts et al., 1999), in combination with studying a dominant-negative form of Rac2 in zebrafish neutrophils *in vivo* (Deng et al., 2011), multiple parallel pathways of Rac2 effector functions have been discovered. Rac2 is required for neutrophil motility and chemotaxis by regulating actin polymerization at the leading edge in a positive feedback loop with phosphoinositide 3-kinase (PI3K) (Yoo et al., 2010). Rac2 is required for adhesion and retention of neutrophils in the hematopoietic tissue, yet not required for their release from this tissue (Deng et al., 2011). In addition, Rac2 is an essential subunit for the phagocyte NADPH oxidase complex, directly interacting with gp19^phox^ and p67^phox^, and is responsible for the generation of superoxide ions during infection (Jonzon and Bindslev, 1991). Furthermore, Rac2 is required for the degranulation of primary granules in neutrophils (Abdel-Latif et al., 2004). It is our expectation that suppressing Rac2 activity in neutrophils will greatly reduce the number of infiltrating neutrophils in the tissue and alleviate patients from over-inflammatory burdens. However, benefits of Rac2 as therapeutic targets have not been previously explored, probably due to the lack of a Rac2-specific inhibitor as well as the fact that Rac2 deficiency results in primary immune deficiency and poor wound healing (Williams et al., 2000).

The zebrafish is a fully sequenced vertebrate model organism with a conserved innate immune system (Deng and Huttenlocher, 2012). The ease of genetic manipulation and the optical transparency of zebrafish larvae made them ideal model organisms to observe the behavior of phagocytes in a non-invasive way and to dissect related molecular mechanisms. Here, we provide the first miRNA that suppresses the expression of Rac2, and demonstrate that partial Rac2 suppression attenuated the acute lethal inflammation under both sterile and non-sterile conditions.

## RESULTS

### miR-722-overexpressing neutrophils are defective in motility and chemotaxis

To test the efficacy of miRNAs as next-generation therapeutics that would restrain neutrophil migration and inflammation, we looked into miRNAs that can suppress *rac2* expression. We performed bioinformatics analysis (TargetScanFish) and identified three miRNAs (miR-194, miR-722 and miR-129) that are predicted to bind to the 3′UTR of both transcript variants of the zebrafish *rac2* gene. miR-722 and miR-129 share the same seed sequence and bind to a perfect seed-matching site in the *rac2* 3′UTR with a context+ score percentile above 90. miR-194 binds to a separate site with a partial seed match and a context+ score percentile of 69, possibly being a weaker regulator of *rac2*. Data compiled from previous miRNA sequencing experiments suggest that miR-722 is intergenic and predominantly produces a mature 3p strand that harbors the *rac2*-binding sequence (www.miRbase.org). In contrast, both the mature 5p and 3p strands of miR-129 are detected, which potentially complicates the biological consequence of overexpressing this miRNA. In addition, the miR-722 level is below the detection limit by quantitative miRNA reverse-transcription (RT)-PCR (RT-qPCR) in sorted neutrophils. Last but not least, the seed-binding sequence is also present in the human *RAC2* 3′UTR. Based upon aforementioned reasons, miR-722 was selected for further characterization.

**Fig. 1.**
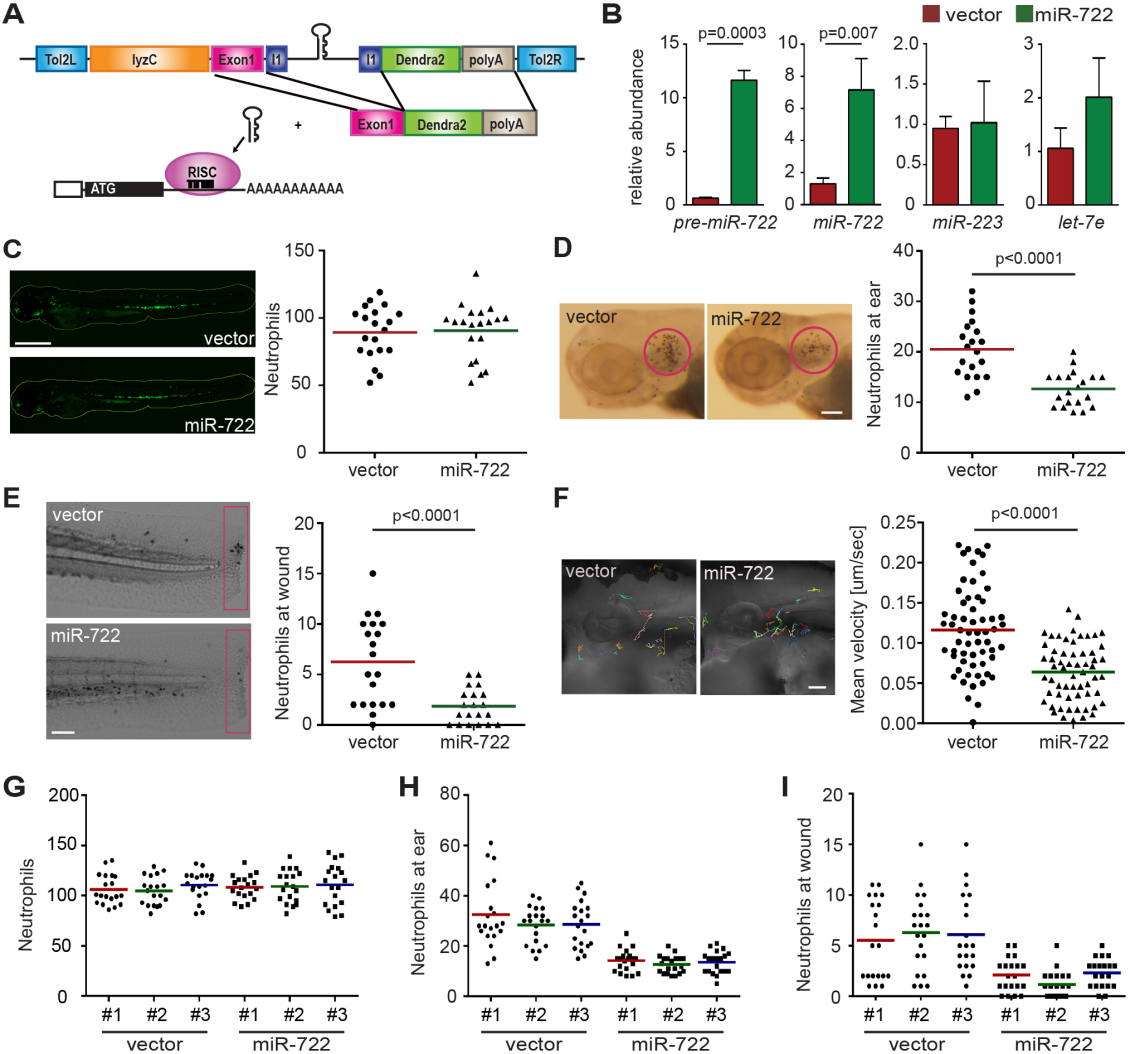
**Neutrophil recruitment and motility is hindered in the miR-722-overexpressing zebrafish line.** (A) A schematic of the Tol2-lyzC:miR-722/Dendra2 plasmid, injected into wild-type AB zebrafish embryos to generate the transgenic line *Tg(lyzC:miR-722/Dendra2)^pu6^* (miR-722). *Tg(lyzC:Dendra2)^pu7^* was generated using the same configuration without the miR-722 insertion (vector). All experiments were performed with F2 larvae at 3 dpf. (B) Relative expression level of precursor and mature miR-722, miR-223 and let-7e (normalized to *U6* expression) in vector and miR-722 lines determined by RT-qPCR. Data are means±s.d. (*N*=3 biological repeats with 10 larvae at each time point in each group). *P* values were calculated with unpaired Student's *t*-test. (C) Representative images and quantification of total neutrophils in vector and miR-722 lines. One representative result of three independent experiments is shown (*n*=20). Scale bar: 500 µm. (D) Representative images and quantification of neutrophil recruitment to localized ear infection in vector or miR-722 larvae at 1 hpi. The infected ear is indicated with the circle. One representative result of three independent experiments is shown (*n*=20). *P* value was calculated with unpaired Student's *t*-test. Scale bar: 100 µm. (E) Representative images and quantification of neutrophil recruitment to tail transection site in vector or miR-722 larvae at 1 h post-injury. Neutrophils in the boxed region were quantified. One representative result of three independent experiments is shown (*n*=20). *P* value was calculated with unpaired Student's *t*-test. Scale bar: 100 µm. (F) Tracks and quantification of neutrophil motility in vector or miR-722 larvae. Results were pooled from three independent larvae (*n*=60). *P* value was calculated with unpaired Student's *t*-test. Scale bar: 50 µm. (G-I) Quantification of total number of neutrophils (G), neutrophils recruited to the ear 1 h post *P. aeruginosa* infection (H) and to the wound 1 h post tail transection (I). One representative result of three independent experiments is shown (*n*=20). No statistical difference among the results from separate founders were observed with unpaired one-way ANOVA.

First, we generated a transgenic zebrafish line that overexpresses miR-722 specifically in neutrophils (schematic in Fig. 1A). To facilitate the identification and characterization of cells expressing this miRNA, a 206 bp genomic DNA sequence flanking miRNA-722 was cloned into an intron that allows co-expression of miR-722 with a green fluorescent reporter protein, Dendra2. Three founders each of the zebrafish that express the vector control or miR-722 were obtained. We observed specific upregulation of both the precursor and mature forms of miR-722 in the transgenic animals, without alterations in the level of miR-223 or a ubiquitously expressed miRNA, let-7e (Fig. 1B), confirming that miRNA biogenesis in neutrophils is intact. In addition, similar numbers of neutrophils were present in both lines (Fig. 1C), indicating that miR-722 does not impair neutrophil biogenesis or survival. We next examined the recruitment of miR-722-overexpressing neutrophils in two separate acute inflammation models: a localized bacterial infection and tail transection. Significantly fewer neutrophils were recruited in miR-722-overexpressing lines in both incidences (Fig. 1D,E; Movie 1). This phenotype was further confirmed in the offspring from separate founders (Fig. 1G-I), excluding the positional effect of the random genomic insertion by the Tol2 transposon method. Furthermore, the motility of the miR-722-overexpressing neutrophils was significantly hampered (Fig. 1F; Movie 2), which phenocopied the Rac2-deficient neutrophils (Deng et al., 2011; Rosowski et al., 2016), coinciding with the prediction that miR-722 downregulates *rac2* expression in neutrophils.

### miR-722 directly suppresses zebrafish *rac2* expression

**Fig. 2.**
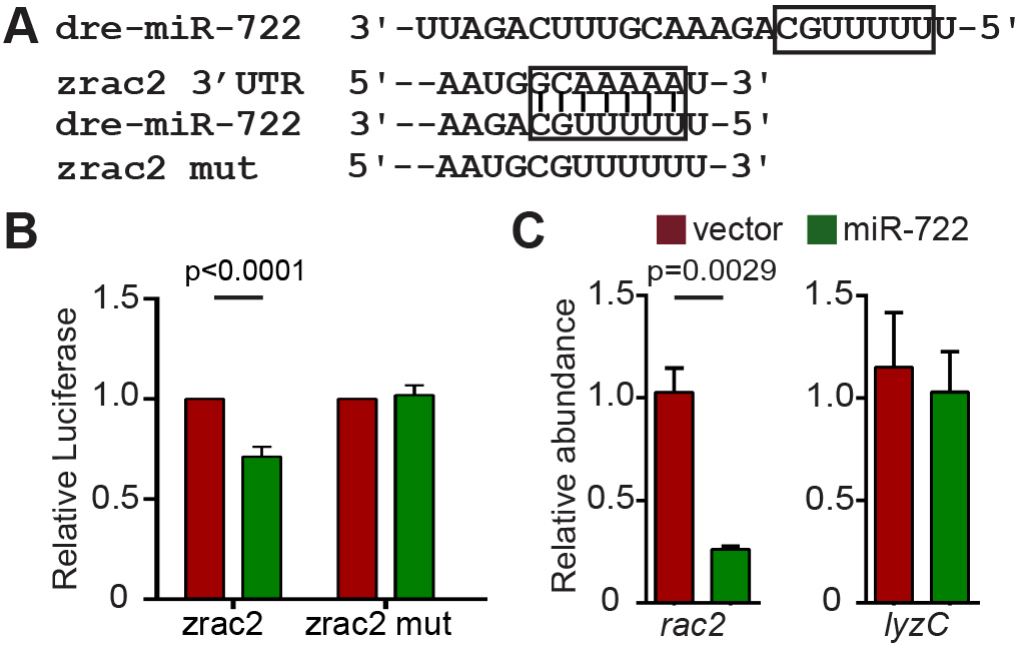
**miR-722 downregulates the zebrafish *rac2* transcript through binding to seed complementary sequences in its 3′UTR.** (A) Sequence of miR-722 and zebrafish *rac2* 3′UTRs. The seed sequence and its binding sites in the 3′UTRs are boxed. (B) Selective suppression of *Renilla* luciferase activity by miR-722 through binding to seed sequence in zebrafish *rac2* 3′UTRs. Results are presented as means±s.d. (*N*=3 independent experiments). *P* values were calculated with paired Student's *t*-test. (C) Relative expression level of *rac2* and *lyzC* mRNA (normalized to *ef1a*) in vector and miR-722 larvae determined by RT-qPCR. Results are presented as means±s.d. (*N*=3 independent experiments with over 20 larvae each/experiment). *P* values were calculated with unpaired Student's *t*-test.

We then confirmed that miR-722 can directly suppress the zebrafish *rac2* gene. The zebrafish *rac2* 3′UTR harbors a miR-722-binding site with perfect seed-sequence match (Fig. 2A). We performed reporter assays to validate the direct translational suppression by miR-722. Expression of miR-722 significantly suppressed the relative luciferase activity, which was dependent on the seed sequences in the zebrafish *rac2* gene (Fig. 2B). Because reporter assays are based on enforced miRNA and transcript overexpression that can yield false-positive results, we measured the endogenous *rac2* transcript level. In the miR-722-overexpressing zebrafish line, the *rac2* mRNA level is significantly reduced (Fig. 2C), suggesting a direct destabilization of the *rac2* transcript by miR-722 in neutrophils. Another neutrophil-specific gene, encoding lysozyme C, was not altered in the same sample, indicating the specificity of miR-722 towards *rac2*.

### Rac2 overexpression rescues miR-722-induced phenotypes

**Fig. 3.**
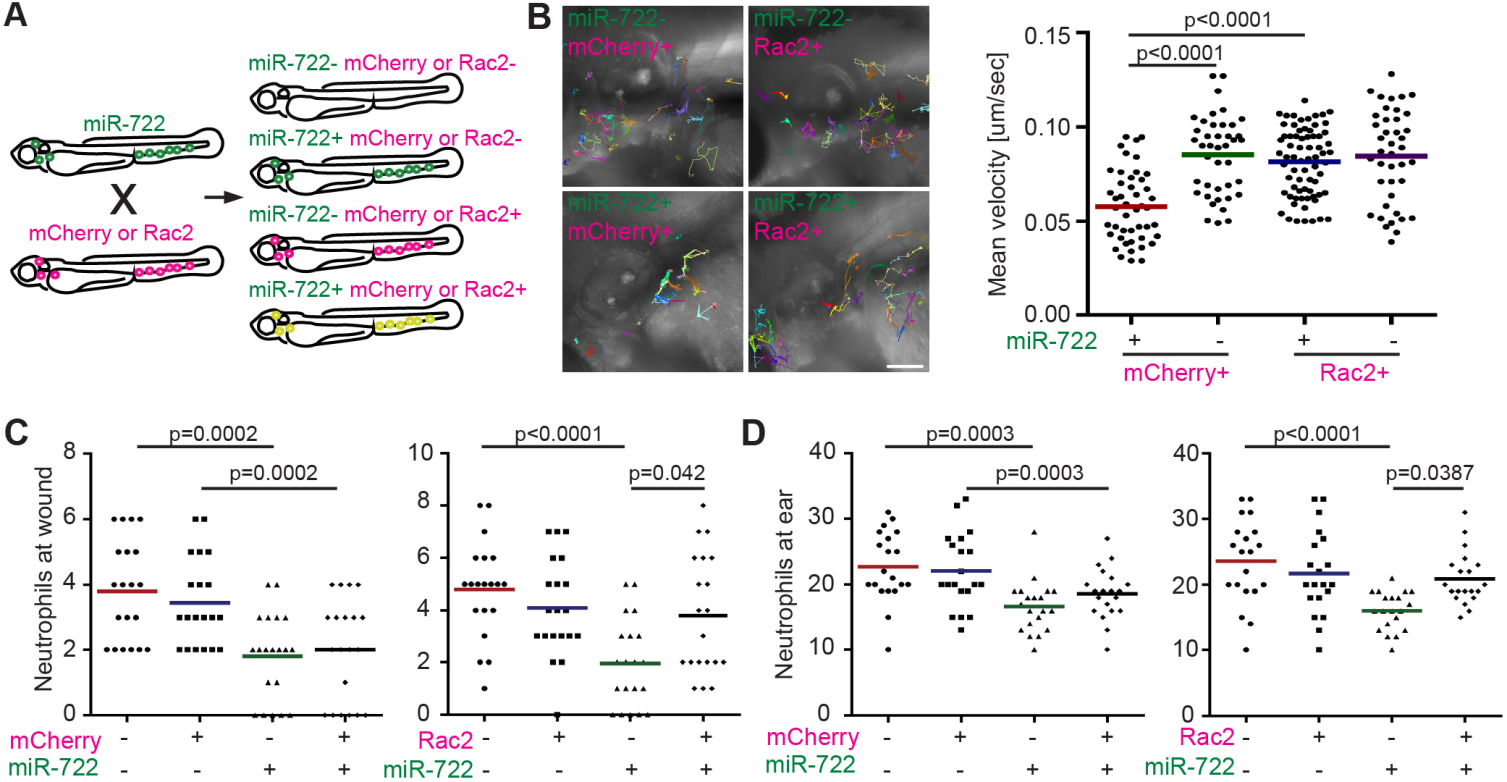
**Overexpression of *rac2* rescues neutrophil recruitment in miR-722-expressing larvae.** (A) *Tg(lyzC:miR-722/Dendra2)^pu6^* (miR-722) was crossed with *Tg(mpx:mCherry-2A-Rac2)* (Rac2) and the offspring were separated into four groups. For control, *Tg(lyzC:miR-722/Dendra2)^pu6^* was crossed with *Tg(mpx:mCherry)* (mCherry). All experiments were performed with F2 larvae at 3 dpf. (B) Tracks and quantification of neutrophil motility in indicated lines. Results were pooled from three independent larvae. *P* values were calculated with unpaired Student's *t*-test. Scale bar: 100 µm. (C,D) Quantification of neutrophil recruitment to tail wounding (C) or localized ear infection (D) in siblings separated into the four groups as depicted in A. One representative experiment of three independent biological repeats is shown (*n*=20 for each group). *P* values were calculated with unpaired two-way ANOVA.

To further validate that *rac2* is a major target of miR-722 in neutrophils, we performed a rescue experiment using a transgenic zebrafish line that overexpresses zebrafish *rac2* followed by the SV40 3′UTR, which is resistant to miR-722-mediated suppression (Deng et al., 2011). A line that expresses mCherry alone was used as a control. Clutch mates were used in this experiment to minimize the impact of genetic variation in different lines (Fig. 3A). Consistent with our data that miR-722 directly downregulates endogenous *rac2* expression, defects in neutrophil motility (Fig. 3B) or their recruitment to tissue injury (Fig. 3C) or infection (Fig. 3D; Movie 3) resulting from miR-722 overexpression were all rescued by *rac2* overexpression, pinpointing *rac2* as a relevant miR-722 target in neutrophils.

### Neutrophil-specific miR-722 overexpression protects zebrafish from lethal systemic inflammation

**Fig. 4.**
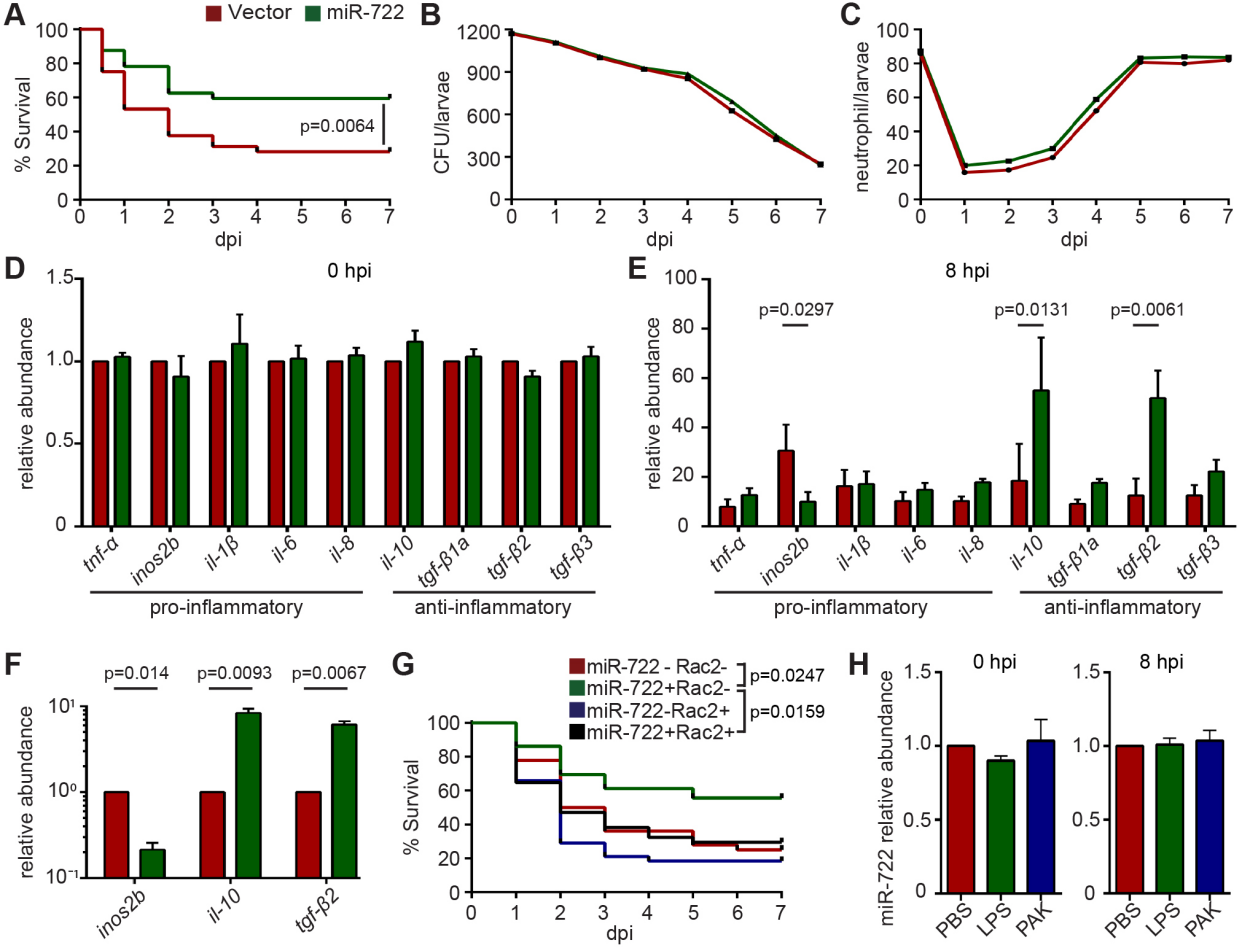
**The miR-722-overexpressing line has increased resistance to bacterial-induced systemic inflammation.** F2 larvae from *Tg(lyzC:miR-722/Dendra2)^pu6^* (miR-722) and *Tg(lyzC:miR-722/Dendra2)^pu6^* (vector) at 3 dpf were injected intravenously with 1000 CFU of *Pseudomonas*. (A) Mortality, (B) CFU and (C) total neutrophil number in the vector or miR-722 lines were documented until 7 days post infection (dpi). (A) One representative experiment of three biological repeats. (B,C) Means±s.d. (*N*=3 biological repeats with 10 larvae at each time point in each group). *P* values were calculated with the Gehan–Breslow–Wilcoxon test. (D,E) Relative abundance of transcripts of pro-inflammatory and anti-inflammatory cytokines to *ef1a* at 0 and 8 hpi. Results are presented as means±s.d. (*N*=3 biological repeats with 20 larvae in each group). *P* values were calculated with one-way ANOVA. (F) Neutrophils were sorted from larvae at 8 hpi and the relative transcript levels of the indicated genes to *ef1a* were quantified. One representative experiment of two independent biological repeats is shown. *P* values were calculated with paired Student's *t*-test. (G) *Tg(lyzC:miR-722/Dendra2)^pu6^* (miR-722) was crossed with *Tg(mpx:mCherry-2A-Rac2)* (Rac2) and the offspring were separated into the four groups as in Fig. 3A. Mortality was documented until 7 dpi. One representative experiment of three independent biological repeats (*n*=20 each group) is shown. *P* values were calculated with the Gehan–Breslow–Wilcoxon test. (H) Relative expression levels of miR-722 before or after intravenous injection with 25 ng of LPS or with 1000 CFU of *Pseudomonas*; means±s.d. (*N*=3 biological repeats with 10 larvae at each time point in each group). No statistical differences were found with unpaired one-way ANOVA.

Neutrophils are a major cell type that causes tissue damage during severe inflammation. Thus, we tested whether miR-722-overexpressing zebrafish were more resistant to lethal inflammatory challenges; in this experiment, we used a bacterial systemic infection model using the Gram-negative bacteria *Pseudomonas aeruginosa* PAK strain*.* The miR-722-overexpressing larvae showed increased survival compared with those overexpressing the vector control (Fig. 4A), despite the presence of similar bacterial burdens (possibly as a result of intact macrophage functions), excluding the possibility that the miR-722-overexpressing line had increased bactericidal activity (Fig. 4B). In addition, in both lines, there was an initial drop of neutrophil number upon infection, which later recovered (Fig. 4C). This increased resistance coincided with a more robust upregulation of the anti-inflammatory cytokines, including IL-10 and the TGF-β family members (Fig. 4D,E). The pro-inflammatory cytokines, including TNF-α, IL-6 and IL-8, were also induced in the miR-722-overexpressing line, but not significantly. Interestingly, *nos2b*, an important pro-inflammatory gene that produces nitric oxide species, was not highly induced. Similar expression changes of *inos2b*, *il-10* and *tgf-β2* were observed in FACS-isolated neutrophils, consistent with neutrophil-restricted overexpression of miR-722 (Fig. 4F). Because zebrafish *nos2b* also harbors miR-722-binding sites, we next examined whether *rac2* overexpression mitigates the protective effect elicited by miR-722. Restoring *rac2* expression in the miR-722-overexpressing line increased susceptibility, to levels comparable to the wild-type larvae, from the acute systemic *Pseudomonas* infection (Fig. 4G), suggesting that miR-722 protects zebrafish from lethal inflammatory challenge via the suppression of *rac2*. In wild-type larvae, the endogenous level of miR-722 was not upregulated during systemic inflammation (Fig. 4H).

**Fig. 5.**
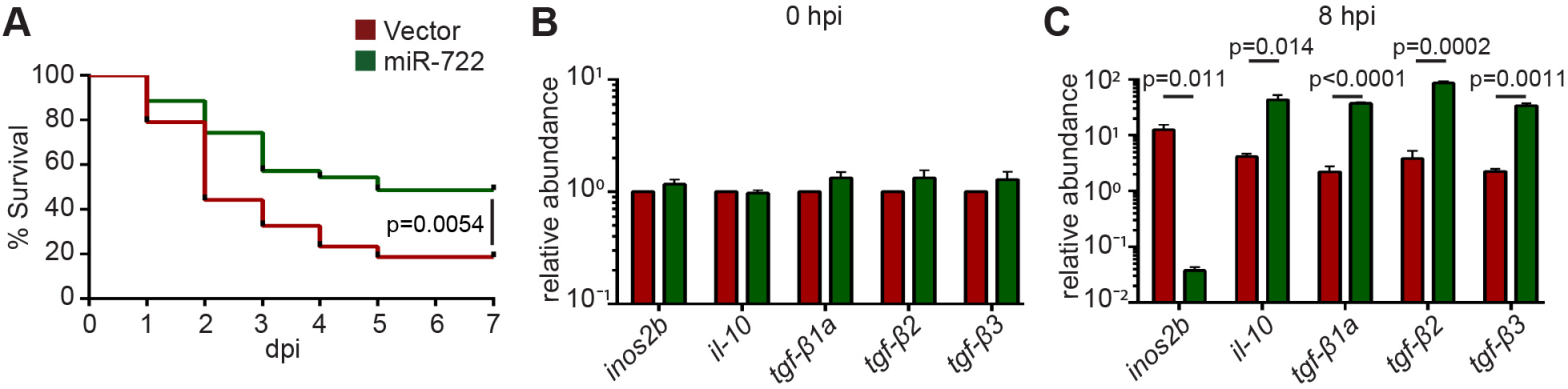
**The miR-722-overexpressing line has increased resistance to sterile systemic inflammation.** F2 larvae at 3 dpf were injected intravenously with 25 ng LPS. (A) Mortality of the vector and miR-722 lines. One representative experiment of three independent biological repeats (*n*=20 each group) is shown. *P* value was calculated with the Gehan–Breslow–Wilcoxon test. (B,C) Relative abundance of transcripts of pro-inflammatory *inos2b* and anti-inflammatory cytokines to *ef1a* at (B) 0 hpi and (C) 8 hpi; means±s.d. (*N*=3 biological repeats with 20 larvae in each group). *P* values were calculated with one-way ANOVA.

We also developed a sterile systemic inflammation model by injecting lipopolysaccharide (LPS) into the zebrafish intravenously. The vector-control-overexpressing larvae succumbed to over-inflammation within 6 days post-injection. In comparison, miR-722-overexpressing larvae survived significantly better (Fig. 5A). Similar to the changes observed with bacterial infection, upregulation of the anti-inflammatory cytokines was also observed with sterile inflammation (Fig. 5B,C).

### miR-722 mimic protects against sterile inflammation

**Fig. 6.**
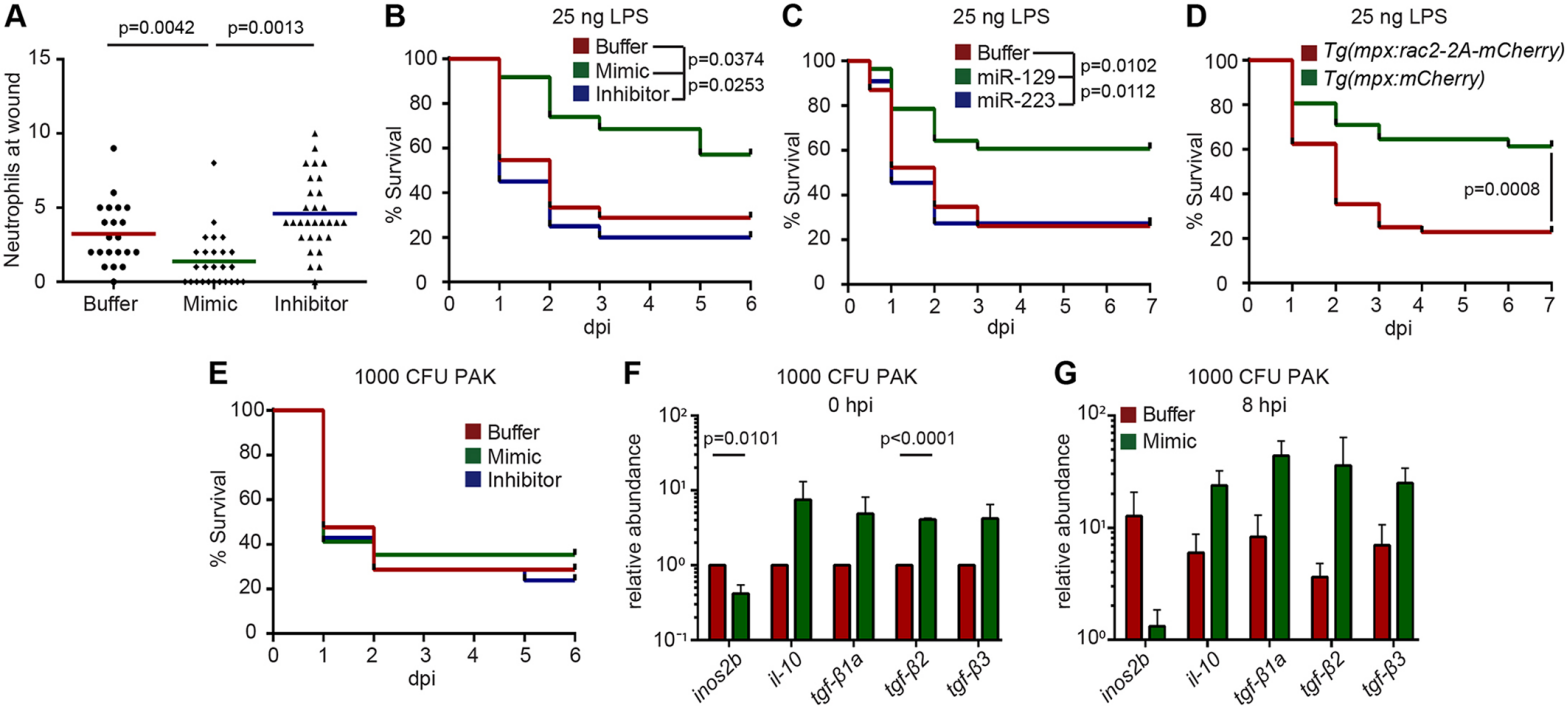
**miR-722 mimic reduces neutrophilic inflammation and mortality from systemic LPS challenge.** Embryos were injected with buffer, miRNA mimic or an miR-722 inhibitor at the one-cell stage and experiments were performed with larvae at 2 dpf. (A) Neutrophil recruitment to the injury site at 1 h post tail transection. One representative experiment of three independent biological repeats is shown (*n*=20 for each group). *P* values were calculated with unpaired one-way ANOVA. (B,C) Survival of larvae with (B) buffer, miR-722 mimic, or inhibitor or (C) buffer, miR-129 mimic or miR-223 mimic upon systemic LPS challenge. One representative experiment of three independent biological repeats (*n*≥20 each group) is shown. *P* values were calculated with the Gehan–Breslow–Wilcoxon test. (D) Survival of *Tg(mpx:mCherry)* and *Tg(mpx:mCherry-2A-Rac2)* larvae with miR-722 mimic upon systemic LPS challenge. One representative experiment of three independent biological repeats (*n*=20 each group) is shown. *P* value was calculated with the Gehan–Breslow–Wilcoxon test. (E) Survival of larvae intravenously injected with PAK at 2 dpf. One representative experiment of three independent biological repeats (*n*=20 each group) is shown. Not significant as determined with the Gehan–Breslow–Wilcoxon test. (F,G) Relative abundance of transcripts of pro-inflammatory *inos2b* and anti-inflammatory cytokines to *ef1a* at (F) 0 h and (G) 8 h after intravenous injection of 1000 CFU PAK. Results are presented as means±s.d. (*N*=3 biological repeats with 20 larvae in each group). *P* values were calculated with one-way ANOVA.

Finally, we injected an miR-722 mimic into zebrafish embryos at the one-cell stage to deliver miR-722 ubiquitously. As expected, neutrophil recruitment to the injury site was impaired in the larvae receiving miR-722 mimic as compared to the buffer-injected larvae, whereas recruitment was more robust in miR-722-inhibitor-injected larvae (Fig. 6A). We observed significantly increased resistance to lethal LPS challenge in the miR-722-mimic-injected larvae compared with buffer- or the miR-722-inhibitor-injected larvae (Fig. 6B), suggesting that miR-722 expression is a potential prophylactic measure in sterile inflammation. miR-129-5p, which is conserved between zebrafish and human, shares the same seed sequence as miR-722. The seed match is present in the 3′UTRs of both the zebrafish *rac2* and human *RAC2* genes. To test a broader translational value of *Rac2*-targeting miRNAs, hsa-miR-129-5p mimic was delivered into the zebrafish embryos. Larvae that received the miR-129 mimic were more resistant to LPS challenge than those receiving a non-*rac2*-targeting miR-223 mimic (Fig. 6B,C). To demonstrate that the miR-722 mimic also elicits its protective role via the inhibition of *rac2* in neutrophils, the mimic was injected into a line that expressed miR-722-resistant *rac2* in neutrophils. Indeed, the protective role of miR-722 was abrogated in the *rac2*-overexpressing line, but not in a line that expresses the mCherry control in neutrophils (Fig. 6D). Taken together, our results suggest that *rac2*-inhibiting miRNA mimics can improve the outcome in sterile inflammation.

By contrast, a protective effect from miR-722 mimic in the *Pseudomonas* sepsis model was not observed (Fig. 6E). Similar amounts of neutrophils were recruited to localized PAK infection (data not shown). In addition, despite the fact that anti-inflammatory cytokines were upregulated in infected larvae that received miR-722 mimic, the fold changes were variable between experiments that did not reach statistical significance (Fig. 6F,G). The different host outcome with miR-722-mimic treatment between sterile and bacterial infection is possibly due to a threshold concentration lower than that of biological relevance or the short-lived effectiveness of the miR-722 mimic to have a sustained effect when battling live organisms that take days to clear (Fig. 4B).

## DISCUSSION

Here, we have identified a miRNA, miR-722, that, when overexpressed in neutrophils, reduces neutrophil chemotaxis and protects the whole organism from both sterile and non-sterile inflammatory assaults. Our findings are of significant importance because we have identified a leukocyte-specific manner to restrain the systemic inflammatory response and have a direct impact on numerous human diseases, including those directly involving an immune component, such as rheumatic arthritis, and those that are not obviously linked, such as diabetes, neurodegenerative disease and cancer.

Here, we have demonstrated that *rac2* is a major target of miR-722 in neutrophils. Rac2 regulates multiple steps in neutrophil-mediated tissue damage, including reducing neutrophil adhesion to the capillary (causing ischemic damage) and neutrophil release of reactive oxygen species and granular contents (major mediators of secondary organ damage). In light of the detrimental roles neutrophils play, it is not surprising that Rac2 inhibition improves the outcome in our systemic inflammation models. It remains to be determined which is the most critical step in this process. Whether it is myelopoiesis, neutrophil exit from bone marrow, chemotaxis, adhesion to blood vessels, degranulation or releasing reactive oxygen or nitrile species requires further investigation. It certainly is possible that multiple steps have to be spontaneously inhibited to elicit a protective effect in treating undesired inflammation. The therapeutic potential of Rac2 inhibition has not been explored previously, probably due to the difficulty in developing a specific chemical inhibitor for Rac2 that would not inhibit the closely related family member Rac1, which is expressed in all cells and is developmentally essential (Duquette and Lamarche-Vane, 2014). Rac1 and Rac2 proteins are very similar in their structure and function, although they harbor different functions in neutrophils (Zhang et al., 2009). miRNAs can bind to the 3′UTR of their target genes and it is very practical to identify miRNAs that target *RAC2* but not *RAC1*, making it possible to selectively suppress *RAC2* expression. We have demonstrated that two different *rac2*-targeting miRNA mimics are equally potent in zebrafish. Many other miRNAs are predicted to target the *RAC2* gene in humans, but not the *RAC1* gene – for example, miR-6090 and miR-6726 (TargetScan) – which warrant further characterization.

It is interesting that *Rac2*-knockout animals or zebrafish expressing a dominant negative form of Rac2 in neutrophils are more susceptible to infections (Deng et al., 2011; Roberts et al., 1999; Rosowski et al., 2016). In contrast, miR-722 overexpression in neutrophils protected zebrafish from lethal challenge of *Pseudomonas* infection. This discrepancy is possibly due to the fact that miRNAs are fine-tuners that modulate the protein expression level post-transcriptionally. In our study, we observed a partial inhibition of *rac2* expression and impaired/delayed neutrophil chemotaxis. We reasoned that neutrophils still preserve some of the effector functions, yet the magnitude of the inflammation and the bystander tissue damage are decreased, which translates into a favorable balance of the pro-inflammatory and anti-inflammatory cytokines that promotes the resolution of inflammation. Along the same line, therapeutic doses and delivery methods of *RAC2*-targeting miRNAs have to be carefully determined in humans to elicit the most favorable outcome.

We have selected a clinical strain of *P. aeruginosa* for our current study because of its prevalence in human sepsis patients (Gotts and Matthay, 2016) and because it is a well-characterized systemic infection model in zebrafish (Clatworthy et al., 2009). *Pseudomonas aeruginosa* is an opportunistic pathogen in humans, not a natural zebrafish pathogen, and it requires a much higher infection dose to cause significant mortality (2000-10,000 CFU) in immune-competent larvae compared with natural fish pathogens, such as *Edwardsiella tarda* (van Soest et al., 2011) and *Streptococcus iniae* (Harvie et al., 2013). It has long been appreciated that neutrophils cause tissue damage while eliminating bacterial infections (reviewed in Weiss, 1989). However, it is difficult to separate these two functions because similar mechanisms, such as reactive oxygen species, proteases and extracellular traps, contribute to both processes. To date, solid evidence that inflammation contributes to mortality in a *Pseudomonas* blood-infection model is not available, although it is an attractive hypothesis based on the literature. Our work has associated the improved survival with increased production of anti-inflammatory pre-resolving cytokines, but not with bacterial burden, providing the first evidence that inflammation is relevant to mortality in this model*.*

There are several recent examples that anti-inflammatory intervention increases zebrafish survival without altering the bacterial burden. Treatment with the IL-1 receptor antagonist anakinra enhanced zebrafish survival in *Shigella flexneri* or *Burkholderia cenocepacia* infection, without affecting the bacterial load (Mazon-Moya et al., 2017; Mesureur et al., 2017). The *myd88*-null mutant also lived significantly longer than wild-type siblings in *B. cenocepacia* infection, with no differences in bacterial burden (Mesureur et al., 2017). In addition to live bacterial infection, we have also used *P. aeruginosa* LPS to induce mortality that is caused by sterile inflammation. We observed similar phenotypes with both live bacteria and a purified bacterial cell-wall component, indicating that miR-772 regulates the inflammation process to favor host survival.

So far, reduction of neutrophil number has not been associated with increased survival in zebrafish infection models. Several primary neutrophil-deficiency models have been established and many are associated with increased susceptibility to infections (reviewed in Harvie and Huttenlocher, 2015). In the WHIM and LAD models, neutrophil recruitment to wounding or infection is completely abolished, indicating that neutrophils provide protective immunity and that a substantial loss of neutrophil function is detrimental to the host.

A partial reduction of neutrophil number can be achieved by disrupting GCSFR/Csf3r signaling, using either a morpholino (Liongue et al., 2009) or a recently generated mutant (Pazhakh et al., 2017), or with the *Escherichia coli* nitroreductase/metronidazole system (Pisharath et al., 2007).

Gcsfr (also known as Csf3r) morphants are more susceptible to *Salmonella* (Hall et al., 2012) and chikungunya virus (Palha et al., 2013). The caveat of this approach – whether Gcsfr depletion affects macrophage number, especially in older larvae – still needs to be determined. In addition, because Gcsfr-depleted larvae were more susceptible to infection than Runx1-depleted larvae (in which both neutrophil and macrophage numbers were reduced) (Hall et al., 2012), it is possible that Gcsfr regulates other aspects of neutrophil biology, not only neutrophil number.

An alternative approach for neutrophil depletion using the nitroreductase/metronidazole system was first performed by Prajsnar et al. (2012), where 50% of neutrophils were depleted without affecting macrophage number. Although the larvae are more susceptible, neutrophils were discovered as a privileged intraphacyte niche for disseminated *Staphylococcus* infection, highlighting the multifaceted role of this phagocyte. In a more recent study, over 95% depletion of neutrophils did not affect zebrafish survival during *Burkholderia* infection (Mesureur et al., 2017). The caveat of this approach is the risk of non-specific alteration in the immune system caused by un-natural phagocyte death.

Nevertheless, neutrophil depletion has been proven to be beneficial in many murine inflammation models, including infections (reviewed in Mócsai, 2013). To be more specific, mice depleted of FcεRI+ neutrophils were less susceptible to experimental cerebral malaria after infection with *Plasmodium berghei*, without reducing the parasite burden in blood (Porcherie et al., 2011). Our research is set apart from the existing literature in that a fine-tuning of neutrophil function, rather than total neutrophil depletion or loss of function, was achieved.

In summary, we have provided a proof-of-concept strategy in treating conditions in which overactivation of the immune system contributes to disease via miRNAs, particularly those targeting *RAC2* expression. Human neutrophils have an estimated circulatory half-life of up to 90 h (Pillay et al., 2010; Tak et al., 2013). Although this measurement may be explained alternatively as the half-life of neutrophil progenitors, a population of older neutrophils survive for several days in the body in other model organisms (Cheretakis et al., 2006; Vincent et al., 1974). miRNA administration in human therapeutic settings could, at least theoretically, be rapid enough to downregulate *RAC2* expression in mature neutrophils and/or long-lasting enough to downregulate *RAC2* during neutrophil maturation in the bone marrow until their mobilization into the circulation. Owing to current technical hurdles that prevent us from effectively delivering miR-772 into neutrophils in the larvae, we have not been able to show the efficacy of miR-722 in treating existing inflammation. Nevertheless, we have demonstrated that miR-722 can be used as a prophylactic measure that alters the overall immune response during systemic inflammation, which may be relevant to conditions in humans, for example as a means to prevent overt inflammation elicited during organ transplantations. With the combination of a yet-to-be-optimized efficient phagocyte-specific delivery system, we provide an alternative concept in restraining unresolving neutrophilic inflammation.

## MATERIALS AND METHODS

### Generation of transgenic zebrafish lines

The zebrafish experiment was conducted in accordance with internationally accepted standards. The Animal Care and Use Protocol was approved by The Purdue Animal Care and Use Committee (PACUC), adhering to the Guidelines for Use of Zebrafish in the NIH Intramural Research Program (protocol number: 1401001018). A 206 bp genomic DNA sequence flanking miR-722 (MI0004765) was PCR amplified using forward: 5′-AATCAGGACTGTGTTGCTGTCT-3′, reverse: 5′-CCTCTTCGTCTTCCTCTCGGC-3′ primers, and inserted into the *Bbs*I site in the intron of the vector modified from De Rienzo et al. (2012). GFP was replaced with Dendra2 and then cloned into the Tol2 backbone containing the lyzC promoter and SV40 polyA. The plasmids were deposited to Addgene (plasmid 97101, Tol2-lyzC-Vector-Dendra2; plasmid 97130, Tol2-lyzC-miR-722-Dendra2). More than three founders (F0) for both *Tg(lyzC:miR-722/Dendra2)^pu6^* and *Tg(LyzC:Dendra2)^pu7^* were obtained as described in the AB background (Deng et al., 2011). Experiments were performed with F2 larvae produced by F1 fish.

### Zebrafish neutrophil recruitment assay

Zebrafish wounding and infection were performed as described (Deng et al., 2011). Hindbrain injection was done as described (Gutzman and Sive, 2009). Briefly, 2 or 3 dpf larvae were amputated posterior to the notochord, or inoculated with *P. aeruginosa* (PAK) into the left otic vesicle or into the ventricle region of the brain at 1000 CFU/embryo. The larvae were fixed in 4% paraformaldehyde at 1 h post-wounding or -infection. Neutrophils were stained with Sudan black and the number at the indicated regions were quantified.

### Dual luciferase reporter assay

Zebrafish *rac2* 3′UTR was amplified with One Step Ahead RT-PCR kit (Qiagen) from zebrafish mRNA using the following primers and inserted into pCS2+GFP using *Eco*RI/*Not*I sites: zRac2+: 5′-GTACAAGTGAGAATTCAGATACACGATTCGTCACTG-3′; zRac2-: 5′-ATTGGCGCCGCGGCCGCCAGTTGTACAGTTTATTTTTGC-3′. Rac2 mutant 3′UTR constructs were generated using Infusion HD cloning kit (Clontech) with the following primers: zRac2 mut+: 5′-TTTTGGCAGAAAATGCGTTTTTTAAACTGTACAACTGGCGGCC-3′; zRac2 mut-: 5′- CATTTTCTGCCAAAAATAATTCCATAC-3′. The mutations were confirmed by sequencing.

The suppression of the reporter expression was measured. Reporter assay constructs were then cloned into psiCHECK2 (Promega) at *Xho*I and *Not*I cloning sites using the following primers to amplify both wild-type and mutant 3′UTRs from pCS2+ constructs. Psi-zRac2+: 5′-TAGGCGATCGCTCGAGAGATACACGATTCGTCACTGT-3′; Psi-zRac2-: 5′-TTGCGGCCAGCGGCCGCCAGTTGTACAGTTTATTTTTGCCA-3′; Psi-zRac2 Mut-: 5′-TTGCGGCCAGCGGCCGCCAGTTGTACAGTTTAAAAAACGCA-3′. miR-722 expression vector was cloned by amplifying the 722 hairpin from the lyzC:miR-722 vector used to create the transgenic line and inserted into pcDNA3.1 at the *Hin*dIII/*Xba*I cloning sites using the following primers: pcDNA-722+: 5′- GTTTAAACTTAAGCTTGCCACCATGGATGAGGAAATCGC-3′; pcDNA-722-: 5′-AAACGGGCCCTCTAGAGACCGGTACCCCCGGGCTGC-3′. Plasmids were deposited to Addgene (plasmid 97158, pSi-check2-zRac2 3′UTR; plasmid 97159, pSi-check2-zRac2 - mut 3′UTR; plasmid 97160, pSi-check2-hRac2 3′UTR; plasmid 97161, pSi-check2-hRac2 - mut 3′UTR; plasmid 97163, miR-722-Dendra pCDNA).

Plasmids were co-transfected into HEK293 cells with Lipofectamine 3000 (Invitrogen). Cells were harvested after 48 h. *Renilla* luciferase activity was normalized with *Photynus* luciferase activity, which were sequentially determined using a dual luciferase reporter assay (Promega) and a plate reader (BioTek). Three independent biological repeats were performed for each 3′UTR.

### Confocal imaging

Larvae at 3 dpf were settled on a glass-bottom dish. Time-lapse fluorescence images were acquired with a laser-scanning confocal microscope (Movie 1; LSM710, Zeiss) with a Plan-Apochromat 20×/0.8 M27 objective. The green and red channels were acquired sequentially with 0.1% power of the 488 nm laser and 0.4% of 561 nm laser, respectively, with a 200 µm pinhole at a speed of 1.27 µs/pixel and averaged (line 2). The fluorescent stacks were flattened using the maximum intensity projection and overlaid with a single slice of the bright-field image. Neutrophil speed was quantified using ImageJ plug-in MTrackJ (Meijering et al., 2012).

### RT-qPCR

Total RNA was purified using the MiRVANA miRNA purification kit (Thermo Fisher Scientific). miRNAs were reverse transcribed with Universal cDNA Synthesis Kit II (Exiqon). miRNA RT-qPCR was performed with ExiLENT SYBR^®^ Green master mix (Exiqon) using LightCycler^®^ 96 Real-Time PCR System (Roche Life Science). Primers used in this study are: miR-223-3p (205986), dre-let-7e-5p (2106780), dre-miR-722 (2107521) and dreU6 (206999). Messenger RNAs were reverse transcribed with Transcriptor First Strand cDNA Synthesis Kit (Roche). RT-qPCR was performed with FastStart Essential DNA Green Master (Roche). Primers are listed in Table S1. All primers amplified a single product according to the melt-curve analysis. The relative fold change is calculated following instructions provided by Real-time PCR Minor with correction of the primer efficiencies (http://ewindup.info/miner/data_submit.htm). A total of 10-20 larvae were used in each repeat to generate an average value that was used to calculate the final mean±s.d. from three independent experiments.

### FACS of dissociated embryo neutrophils and one-step RT-qPCR

Larvae at 3 dpf from *Tg(lyzC:miR-722/Dendra2)^pu6^* and *Tg(LyzC:Dendra2)^pu7^* were injected with 1000 CFU *P. aeruginosa* (PAK) into the tail vein and incubated to 8 hours post-infection (hpi). Neutrophils were sorted out by FACSARIA II with the 488 laser as described (Deng et al., 2011). Neutrophil RNA was extracted as described above and one-step RT-qPCR was performed with SuperScript^®^ III Platinum^®^ SYBR^®^ Green One-Step qRT-PCR Kit (Invitrogen), using LightCycler^®^ 96 Real-Time PCR System (Roche Life Science).

### Survival assay

Larvae at 3 dpf were injected with 1 nl of 25 ng/nl LPS or 1000 CFU *P. aeruginosa* (PAK) into the tail vein and incubated individually in 96-well plates. Survival was tracked for 7 days or when one group reached 100% mortality. Representative experiments of at least three independent repeats (*n*≥20 larvae in each experiment) were shown.

### miRNA mimic and inhibitor delivery

All mimics and the miR-722 inhibitor were synthesized by Thermo Fisher Scientific. Embryos at the one-cell stage were injected with 1 nl of 15 µM dre-miR-722 mimic (#4464066), dre-miR-722 inhibitor (#4464084), hsa-miR-129-5p mimic (#4464084), 1 µM dre-miR-223 mimic (#4464066) or buffer as a control. Tail wounding and survival assays were carried out as described above but at 2 dpf.

### Statistical analysis

Statistical analysis was carried out by PRISM 6 (GraphPad). Unpaired Student's *t*-test (comparing two groups), one-way ANOVA (when comparing to single group), or two-way ANOVA (for multiple comparisons) were utilized in neutrophil recruitment assays and the reporter assays. For RT-qPCR, each gene was normalized to the reference gene and compared with paired Student's *t*-test. For survival assays, the Gehan–Breslow–Wilcoxon test was performed with a log-rank test and confirmed with Kaplan–Meier curve to ensure compatibility.

## Supplementary Material

Supplementary information

First Person interview
