# Supplementary material for: Overexpression of microRNA-722 fine-tunes neutrophilic inflammation by inhibiting Rac2 in zebrafish
Source: Dis Model Mech. 2017 Nov 1;10(11):1323–32. doi: 10.1242/dmm.030791 (PMC5719257; doi:10.1242/dmm.030791)
Supplement: Supplementary information [file dmm-10-030791-s1.pdf]

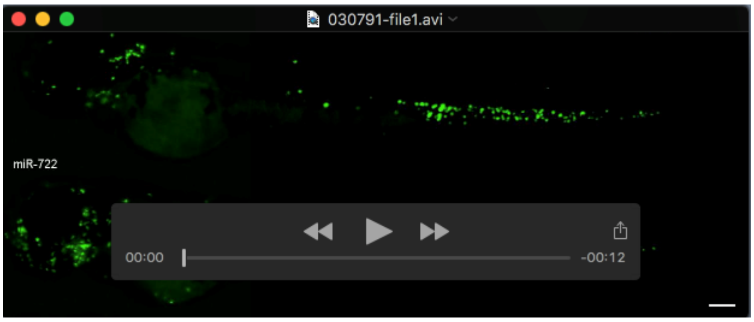

Movie S1. Neutrophil recruitment to ear infection in vector and miR-722 larvae.

Lateral view of larvae with neutrophils expressing vector or miR-722, responding to a localized ear infection. Delayed recruitment of neutrophils were observed in the miR-722 line. Scale bar: 100 µm.

Disease Models & Mechanisms • Supplementary information

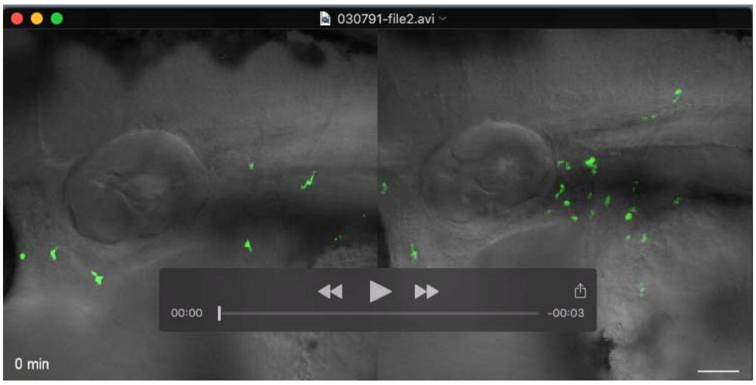

Movie S2. Neutrophil random motility in vector and miR-722 larvae.

Lateral view of neutrophil random migration in the mesenchymal tissues of the head. Note reduced proportion and speed of neutrophil migration in miR-722 larvae. Scale Bar: 50 µm.

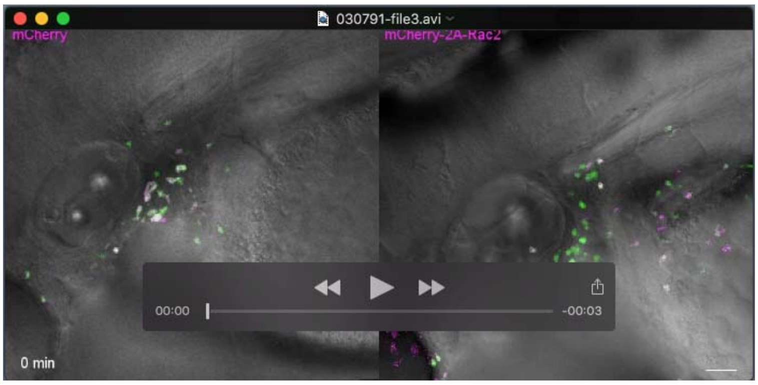

Movie S3. Rac2 rescues the motility defect in the miR-722 larvae.

Lateral view of neutrophil random migration in the mesenchymal tissues of the head. Note the increased proportion and speed of larvae expressing Rac2, but not the mCherry control, in the miR-722 background. Scale Bar: 50 µm.

Table S1. Primers used for RT-qPCR

Gene	Transcripts AN#	Forward	Reverse
dre-Rac2	ENSDART00000055407.3	5'-ggctgcgtgatgctctaaa-3'	5'-tgttggtttataggagaaaaccagt-3'
dre-pre722	ENSDART00000118986.1	5'-cggagtggaatttgaaacgttttggc-3'	5'-cggagcgaaatctgaaacgtttctgc-3'
dre-LyzC	ENSDART00000080549.3	5'-gggattctccattggcaac-3'	5'-ctcggtgggtcttaaacctg-3'
dre-TNF-α	ENSDART00000025847.8	5'-cagagttgtatccacctgtta-3'	5'-ttcacgctccataagaccca-3'
dre-NOS2b	ENSDART00000019431.8	5'-aacggcatcatgaactgttg-3'	5'-tacattgtagtcctccatgcaaa-3'
dre-TGF-β1a	ENSDART00000060839.2	5'-atgctgtgtacccgcaatc-3'	5'-gtgttgccttcccacgtaat-3'
dre-TGF-β2	ENSDART00000148927.1	5'-cgagccctggatactgctt-3'	5'-tcgatgtagagcgagcgtaa-3'
dre-TGF-β3	ENSDART00000019766.8	5'-cagtgctgacaccacacaca-3'	5'-gcccacgtagtagaggatgg-3'
dre-IL-1β	ENSDART00000169225.1	5'-ggctgtgtgtttgggaatct-3'	5'-tgataaaccaaccgggaca-3'
dre-IL-6	ENSDART00000166112.1	5'-tcaacttctccagcgtgatg-3'	5'-tctttccctcttttcctcctg-3'
dre-IL-8	ENSDART00000161996.1	5'-tttacagtgtgggcttggaggg-3'	5'-gcgtcggctttctgtttca-3'
dre-IL-10	ENSDART00000110673.1	5'-aagcgggatatggtgaaatg-3'	5'-gacccccttttccttcatct-3'
dre-Ef1a	ENSDART00000023156.6	5'-tgccttcgtcccaatttcag-3'	5'-taccctccttgcgctcaatc-3'

Disease Models & Mechanisms • Supplementary information
