# Supplementary material for: Overexpression of microRNA-722 fine-tunes neutrophilic inflammation by inhibiting Rac2 in zebrafish
Source: Dis Model Mech. 2017 Nov 1;10(11):1323–32. doi: 10.1242/dmm.030791 (PMC5719257; doi:10.1242/dmm.030791)
Supplement: First Person interview [file dmm-10-030791-s2.pdf]

© 2017. Published by The Company of Biologists Ltd | Disease Models & Mechanisms (2017): doi:10.1242/dmm.030791: Supplementary information

FIRST PERSON

First person – Alan Hsu

First Person is a series of interviews with the first authors of a selection of papers published in Disease Models & Mechanisms, helping early-career researchers promote themselves alongside their papers. Alan Hsu is first author on 'Overexpression of microRNA-722 fine-tunes neutrophilic inflammation by inhibiting *Rac2* in zebrafish', published in DMM. Alan is a PhD student in the lab of Qing Deng at Purdue University, IN, USA, investigating the role of microRNA in innate immune cells and its effect on host–pathogen interactions and immune dynamics.

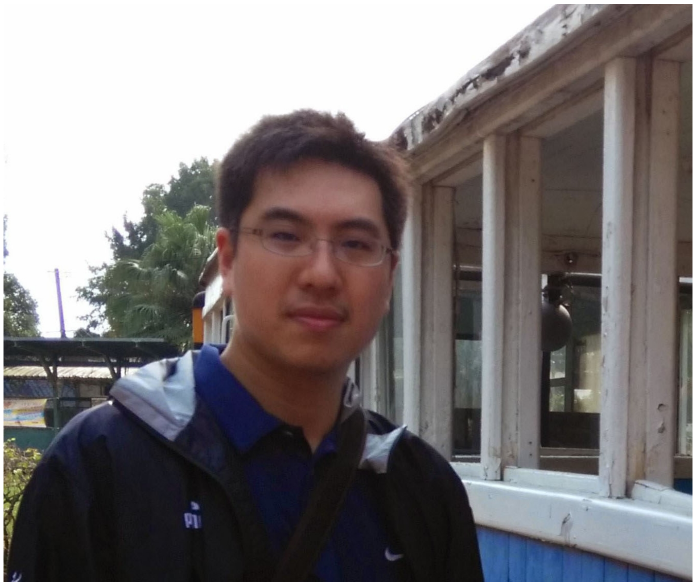

Alan Hsu

How would you explain the main findings of your paper to non-scientific family and friends?

Control of the immune response is a delicate balance of activation to combat foreign pathogens, and keeping it in check to avoid damage to the host itself. Thus, maintaining just the right amount of immune activation is a difficult and delicate task. As such, the microRNAs (miRNAs) that serve as fine-tuners and moderate regulators of virtually all cellular processes seemed an appealing research target. We identified a novel miRNA, miR-722, which is expressed in low quantities in neutrophils, a type of immediate response innate immune cell. We then generated a zebrafish line that overexpressed miR-722 – tagged with a fluorescent protein – specifically in neutrophils. These fish demonstrated lower neutrophil recruitment to a tail wound and regional infection site, but exhibited improved survival rates during systemic infection and sterile inflammation. Diving into the mechanism, we found that miR-722 specifically targets and downregulates Rac2, a Rho-GTPase protein that has been shown to be important for cell motility and migration, and which can explain the decrease of neutrophil recruitment. To summarise, when we stimulated an immune response in fish, we found that overexpression of miR-722 in neutrophils can regulate inflammation and lessen the unwanted effects of over-inflammation.

What are the potential implications of these results for your field of research?

Our goal was to identify miRNAs that are not normally expressed in neutrophils and introduce them in order to identify potential regulators of immune recruitment and function. In science there is a great satisfaction in approaching a question with an unprecedented strategy and then deciphering the underlying biological mechanism.

What are the main advantages and drawbacks of the model system you have used?

Zebrafish is a fully sequenced model organism and its innate immune system is similar to that of humans, making it a good model to study acute inflammation scenarios. In addition, zebrafish larvae are transparent, making real-time imaging much easier and accessible for the study of immune cell properties and behaviour. Furthermore, the zebrafish is easy to genetically edit and the passage time is relatively short. However, although the innate immune system is over 98% conserved between zebrafish and humans, there are some receptors or immune components that may be different as zebrafish live in water and have unique methods of dealing with pathogens that humans would not normally encounter.

"In science there is a great satisfaction in approaching a question with an unprecedented strategy and then deciphering the underlying biological mechanism."

What has surprised you the most while conducting your research?

It was a bit counterintuitive at first that hindering neutrophil recruitment (by overexpressing miR-722) during a systemic inflammation challenge improved survival rates, as you would think the pathogen would need to be cleared by these innate immune cells where neutrophils are the first line of defence to reach the inflammation site. We thus hypothesised that it was due to us only expressing miR-722 in neutrophils where the other innate immune cells were intact. When miR-722 was introduced into the whole embryo, survival rates were comparable during systemic bacterial challenge, but further improved during sterile inflammation. A closer look shed light on the mechanism; iNOS was lowered in neutrophils while anti-inflammatory cytokines were upregulated, suggesting that miR-722 induces a less activated state and thus may protect against undesired inflammation effects.

Alan Hsu's contact details: Department of Biological Sciences, Purdue University, West Lafayette, IN 47907, USA.

Email: hsu116@purdue.edu

Disease Models & Mechanisms

Disease Models & Mechanisms (2017): doi:10.1242/dmm.030791: Supplementary information

Describe what you think is the most significant challenge impacting your research at this time and how will this be addressed over the next 10 years?

The field of miRNA research is still blooming as more and more miRNAs are identified and characterised, and knowledge of the cellular processes in which they are involved is expanding as well. However, not all miRNAs are conserved between different organisms even though the nomenclature may be the same. Furthermore, the binding sites of miRNAs on the target mRNA may also not be conserved, leading to challenges in target validation and miRNA and target mRNA tissue expression profiles. Given the advancements and throughput of sequencing and methods to identify miRNA–mRNA hybrids, we hope that one day we may have an easy and accessible method to identify and validate miRNA–mRNA interactions, instead of relying on bioinformatics for target prediction.

What's next for you?

My current work focuses on the dynamics and interactions of miRNAs in innate immune cells and how miRNAs affect immune cell behaviour and host–pathogen interaction. In the immediate future I will work to characterize more miRNAs and investigate their effects in neutrophils and macrophages. After identifying potential miRNA candidates, we may expand our work into the study of various pathogens, and how innate immune cell interactions affect infection outcome.

Reference

Hsu, A. Y.-H., Wang, D., Gurol, T., Zhou, W., Zhu, X., Lu, H.-Y. and Deng, Q. (2017). Overexpression of microRNA-722 fine-tunes neutrophilic inflammation by inhibiting *Rac2* in zebrafish. *Dis. Model. Mech.* **10**, doi:10.1242/dmm.030791.

Disease Models & Mechanisms
